# The Prevalence of Specific Phobia by Age in an Italian Nationwide Survey: How Much Does it Affect the Quality of Life?

**DOI:** 10.2174/1745017901915010030

**Published:** 2019-02-20

**Authors:** Federica Sancassiani, Ferdinando Romano, Matteo Balestrieri, Filippo Caraci, Guido Di Sciascio, Filippo Drago, Maria Carolina Hardoy, Maria Francesca Moro, Rita Roncone, Martina Piras, Antonio Preti, Liliana Dell’Osso, Carlo Faravelli, Mauro Giovanni Carta

**Affiliations:** 1 University of Cagliari,Cagliari,Italy; 2 University of Rome “La Sapienza”,Rome,Italy; 3 Psychiatry Unit, DAME, University of Udine,Udine,Italy; 4 University of Catania,Catania,Italy; 5 I.R.C.C.S. Associazione Oasi Maria SS. ONLUS-Troina, Troina, Italy; 6 University of Bari, Bari, Italy; 7 Mailman School of Public Health Columbia University,Columbia, NY, US; 8 University of L’Aquila,L’Aquila,Italy; 9 University of Pisa,Pisa,Italy; 10 University of Florence,Florence,Italy

## Abstract

**Introduction:**

The study aimed to see if a community survey conducted by clinical interviewers with semi-structured psychiatric interviews shows lifetime prevalence rates of Specific Phobia (SP) similar to those found by surveys carried out by lay interviewers and if the high level of impairment found in SP may be confirmed.

**Methods:**

This is a community survey on an Italian nationwide sample randomly selected from registers of municipalities. Tools: semi-structured ANTAS psychiatric interview derived from the SCID-DSM-IV, carried out by clinicians (psychologists or physicians); Short Form Health Survey (SF-12) as a measure of Quality of Life (QoL). Analyses: means of the *χ*^2^ test odds ratios were adopted to test several associations regarding SP prevalence. One-way ANOVA was used to compare different groups on attributable burden due to SP and/or other disorders in worsening QoL.

**Results:**

The lifetime prevalence of SP was 2.3%. No difference was found by age class. Females showed more than twice the frequency of males (*p*<0.0001). The disorders showing the closest association with SP were: social phobia (OR=17.53); general anxiety disorder (OR=11.57); anorexia (OR=11.13) and agoraphobia (OR=10.03), but also obsessive compulsive disorders (OR=8.8), eating disorders (OR=7.2), panic disorder (OR=5.9), post-traumatic stress disorder (OR=5.8), and major depressive disorder (OR=4.8) presented an association that achieved statistical significance. The QoL of people with SP and at least one disorder of anxiety, mood or eating in comorbidity, measured as a score at SF12, was worse than controls without SP (*p* <0.001) but that of people with SP without co-morbidity was not (*p* = 0.809).

**Conclusion:**

An epidemiological study conducted by clinical interviewers through semi-structured interviews appears to re-dimension the impact of SP, at least from the public health perspective. Future prospective studies will better clarify the role of SP in the context of anxiety disorders.

## 1 INTRODUCTION

Although Specific Phobia (SP) is a topic not often addressed by epidemiologists [1], we have today a series of studies that place the lifetime prevalence of this disorder from 7.4% to 12.5% [2-10]. A milestone was the World Health Organization’s World Mental Health Survey, an epidemiology project conducted on 25 representative population-based samples coming from 22 countries and on 124.902 interviews in total [10]. This large survey has found overall cross-national lifetime SP prevalence rates of 7.4%, with higher frequencies in females (9.8 **vs.*.* 4.9%) and in high- and higher-middle-income countries [10].

Large-scale epidemiological studies have established that specific phobia is associated with a high level of disability and co-morbidity with other psychiatric disorders [7]. In fact, the National Comorbid Survey shows a considerable impairment in people having a diagnosis of SP, with 34.2% reporting significant role impairments, even higher than in agoraphobia (26.5%), and similar to social phobia 33.5% [11]. This shows that comorbid phobias are more severe and impairing than phobias without comorbidity [11].

Based on the results of epidemiological studies, it is commonly accepted that the onset of phobia is precocious and precedes that of other psychiatric disorders in comorbidity [10]. However, one of the few studies conducted through repeated interviews on the same cohort showed a cumulative incidence that increased up to 50 years (age of last evaluation), with a prevalence in the cohort that increased fivefold from 1978 to 2008 [12]. These results appear to indicate that SP onset is not restricted to youth groups. It is to be noted that the latter research differs from others because in this survey, the diagnoses were conducted by clinicians through highly structured interviews and not by lay interviewers.

It is discussed whether a methodology adopting lay interviewers and rigid structured interviews can identify cases that overlap with those that would be recognized in a clinical setting [13-15]. In fact, the diagnoses derived from non-clinical interviewers might have identified a broader spectrum of cases (including sub-threshold ones) than those known in clinical practice. Although clinical reappraisal studies show sufficient concordance between structured interview-based and clinical diagnoses of specific phobia [16], the methodology of such reappraisal studies has raised relevant critical issues [15].

In contrast, the methodology based on structured interviews and the collection of all symptoms by lay interviewers may provide information about prevalence and relevance of sub-syndromic pictures [17]. The results of epidemiological surveys in the community and primary care settings adopting such a methodology have in fact clarified that there is an impact in terms of disability associated with sub-syndromic features in several disorders [18]. Such results appear to indirectly confirm the validity of the approach: if these conditions are not clinically confirmed, they should not be associated with disability. But in the case of SP, the question arises as to whether comorbidities with other clinically relevant and severely disabling pathologies (which is very frequent) may lead sub-threshold cases of SP to apprear as an association with disability that may not be caused by the SP per se but by the conditions in co-morbidity. It is, therefore, necessary to clarify to what extent the levels of impairment found in people with social phobia depend on this disorder, to what extent they are the consequence of comorbid disorders and how much they may not depend on the amplifying effect of multiple concomitant disorders.

We consider this of interest to study how much SP may impair the perception of Quality of Life (QoL). This concept involves physical and psychological components of wellness [19]. Thus, QoL is used as an outcome measure in chronic diseases and in those that require long-term treatments and which greatly impact daily life [20-22].

Our study, therefore, intends to see if a community survey conducted by clinical interviewers with semi-structured psychiatric interviews will show lifetime prevalence rates of SP similar to those found in surveys carried out by lay interviewers and if the high level of impairment found in SP is confirmed.

## 2 METHODS

The methodology of the epidemiological study has been described in detail in a previously published paper [23].

### 2.1 Design

It is a community epidemiologic survey.

### 2.2 Setting

The survey was conducted in six Italian regions (Abruzzi, Friuli-Venezia Giulia, Puglia, Sicily, Sardinia and Tuscany) representative of the geographical distribution and economic diversity of the 20 Italian regions. In each region, one urban, one suburban and at least one rural area were chosen.

### 2.3 Sample

The sample was randomly selected from the records of the municipalities involved. Randomization was conducted after stratification in eight cells by gender and age groups 18-29; 30-44; 45-64; >64. People selected were contacted by phone and email. The interviews were conducted face-to-face. Diagnoses were conducted according to DSM-IV using a semi-structured interview (ANTAS-SCID-IV) carried out by physicians or clinical psychologists [23]. The Italian version [24] of the Short Form Health Survey (SF-12) [25] was used to measure the QoL.

### 2.4 Statistical Analysis

The measure of frequency adopted was lifetime prevalence.

The odds ratio for DSM-IV simple phobia lifetime prevalence by age, gender and co-morbidity with DSM-IV anxiety, mood and eating disorders diagnosis was calculated using one group for each table as a “pivot”.

The specific frequencies and the measure of association were calculated in people <30 years old **vs.*.* ≥30 years old; these were calculated concerning any anxiety, mood or eating disorder in comorbidity as well as for comorbidity with Major Depressive Disorder (MDD), Panic Disorder (PD) and Generalized Anxiety Disorder (GAD).

Statistical significance in these analyses was calculated by means of the *χ*^2^ test odds ratios as the measure of association and confidence intervals at 95% by adopting Miettinen’s simplified method [26].

The numeric comparisons between the scores of SF-12 of the different groups as well as the comparison of the value of attributable burden derived from the SF-12 in different groups were made by means of One-way ANOVA.

The “attributable burden” due to SP in worsening QoL was calculated as the difference between the SF-12 score in a sample of people without SP and the SF-12 score of the sample with SP. This control group of people without SP was randomly extracted in a stratified (by gender and age) block created for each person with SP, including all people without SP of the same gender and ±2 years old from the database of the research.

A similar matching after block (by gender and age) was used to calculate the weight of the SP in worsening the QoL in disorders with more frequent comorbidity (MDD, PD and GAD). That is to say, a group of persons with that specific disease (MDD, PD and GAD) was made, but without the SP, from the database matching 1/2 with each person with this specific disorder plus SP. The attributable burden was calculated as the difference between the SF12 score of people with the disorder (MDD, PD or GAD) and without SP and the SF-12 score of people with the same disorder with SP.

### 2.5 Ethical Aspects

The researchers provided full information about the aims of the research, about the possibility of terminating the interview and data protection and privacy laws to each interviewed person. The researchers explained that the data would be used as an anonymous database to guarantee confidentiality. Each participant signed a written informed consent form. Each step of the study was carried out in accordance with the Helsinki Declaration.

## 3 RESULTS

The lifetime prevalence of SP in the overall sample was 2.3%. No variation of frequencies by age class emerged, but females had more than twice the frequency of males (3.17 *vs.*. 1.18%, *p* <0.0001) Table **1**. GAD (18.5%), PD (16.6%) and MDD (16.6%) were the most common in people with SP between anxiety, mood or eating disorders Table **2**. However, in relation to the frequencies in people without SP, the disorders showing the closest association with SP were: Social Phobia (OR=17.53); GAD (OR=11.57); Anorexia (OR=11.13) and Agoraphobia (OR=10.03), but also Obsessive Compulsive Disorders (OR=8.8), Eating Disorders (OR=7.2), Panic Disorder (OR=5.9), PTSD (OR=5.8), Major Depressive Disorder (OR=4.8) presented an association that achieved statistical significance Table **2**.

People with SP under 30 years of age did not show a higher frequency of at least one diagnosis (anxiety, mood or eating disorder) in comorbidity compared to people over 29. The same occurred for comorbidity with MDD, PD and GAD Table **3**.

The QoL of people with SP was worse than that of controls without-SP matched by gender and age (*p* = 0.005).

The QoL of people with SP and at least one comorbid of anxiety, mood or eating disorder measured as a score at SF12, was worse than controls without SP (*p* <0.001) but of people with SP without co-morbidity, it was not worse (*p* = 0.809) Table **4**.

The attributable burden in worsening QoL due to SP without comorbidity with anxiety mood or eating disorders was found lower than all the psychiatric disorders (MDD, PD, Eating Disorders, PTSD and OCD) and all non-psychiatric diseases (Celiac Disease, Multiple Sclerosis, Wilson’s Disease and Carotid Atherosclerosis) Table **5**. On the contrary, attributable burden in worsening QoL due to SP in comorbidity with anxiety mood or eating disorders was found similar to that of diseases such as Wilson’s Disease and Carotid Atherosclerosis and to psychiatric disorders such as MDD, PD, Eating Disorder, PTSD and OCD; it was found higher than the attributable burden due to Celiac Disease and Panic Disorder and lower only than a severe and disability-associated disease such as Multiple Sclerosis Table **5**. The difference between attributable burden in worsening QoL due to SP with comorbidity with anxiety mood or eating disorders and the attributable burden due to SP without comorbidity was of statistical significance.

Table **6** shows how the association of SP in co-morbidity worsens the QoL of people with MDD, PD and GAD to a statistically non-significant extent. In fact, the average scores at SF-12 in samples of people in the general population with these diagnoses but without SP were higher than those of people in our sample with these diagnoses in comorbidity with SP. However, in none of the three cases did the comparison reach statistical significance (MDD 33.0 ± 9.1 with SP *vs.* 35.5 ± 7.7 without SP, *P*=434; PD 33.3 ± 9.8 with SP *vs.* 34.55 ± 6.2 without SP, *P*=0.700; GAD 30.9 ± 7. with SP *vs.* 32.7 ± 7.5 without SP, *P*=0.560).

Even the attributable burden due to SP in the three sample of people with MDD, PD and GAD in comorbidity with SP was found lower than that due to SP comparing people with SP to those without. Thus, the impact of SP was found to be similar in people with MDD, PD and GAD and in people without such a diagnosis.

## 4 DISCUSSION

An epidemiological survey conducted on a national basis in Italy with diagnostic methods closer to clinical practice (clinical interviewers and use of semi-structured interviews) than those usually adopted in community surveys (lay interviewers and structured interviews) has found lower (2.3%) lifetime prevalence rates of SP in the community compared to the range found in surveys conducted with a different methodology (7.4-12.5%).

Women confirmed frequencies of 2.5 times higher than males, but unlike other studies, the frequencies were found stable in different age groups. This datum is consistent with the result of the Zurich study [12] which showed a stable incidence rate in different ages; it must, in fact, be considered that the average duration of the disorder is not all life-long but about 20 years, thus a stable incidence rate (and an increase in the cumulative rate over the years) may produce a stable lifetime prevalence. The Swiss study is the only one, like ours, that employed clinical interviewers [12].

The study confirms a close association of SP with other psychiatric disorders, in particular, anxiety disorders, especially Social Phobia, Agoraphobia, Generalized Anxiety Disorder and Panic Disorder, but also with other psychiatric disorders such as Anorexia and Major Depressive Disorder. The frequency of co-morbidity with Major Depressive Disorder is slightly lower than that reported in the WMH survey, while the association with anxiety disorders does not appear to show differences [10]. The frequency of co-morbidities with other disorders does not differ under 30 years, and also not for the association with at least one diagnosis of mood, anxiety or eating disorders.

Our study did not evaluate the age of onset of SP or other comorbid disorders. However, our data on the stability of the prevalence of SP in the different age groups, as well as the stability of frequency of co-morbid disorders between young and elderly people did not appear to support the hypothesis that SP can be considered a frequent antecedent insurgent in youth always of other disorders, especially anxiety disorder that would appear later.

The impact produced by the disorder on the QoL was found to be considerable, but on analyzing the data in detail, this impact was found to be much higher in the forms of SP in comorbidity with other disorders than in the forms in which SP is present as the only psychopathological element. In this case, the burden due to disorder in worsening the QoL is quite low. This was also evidenced by the fact that the attributable burden in worsening QoL due to non-comorbid SP was significantly lower than that of all other compared disorders, both psychiatric (MDD, PD, Eating Disorders, Agoraphobia, PTSD) as well non-psychiatric (Multiple Sclerosis, Wilson's Disease, Celiac Disease and Carotid Atherosclerosis). On the contrary, the attributable burden in worsening QoL due to SP with comorbidity with anxiety mood or eating disorders was found similar to that of diseases such as Wilson’s Disease and Carotid Atherosclerosis and to psychiatric disorders such as MDD, PD, Eating Disorder, PTSD and OCD; the burden was found higher than the attributable burden due to Celiac Disease and Panic Disorder and lower only than a severe and disability-associated disease such as Multiple Sclerosis. Although in MDD, PD and DAG, the association with SP appears to aggravate the QoL, this aggravation is not worse than that caused by the same disorders when not in comorbidity with SP. These results appear to put into perspective the statement that SP is associated with a substantial impairment [10, 11].

In conclusion, an epidemiological study conducted by clinical interviewers through semi-structured interviews appears to re-dimension the impact of a disorder such as SP, at least from the public health perspective, owing to:

1. the frequency of the disorder in the community, so its impact in terms of public health appears to be lower than the estimate using lay interviewers and structured interviews for diagnosis;
2. the burden in worsening the QoL attributable to the disorder is very low if it excludes the sole effect of the forms in comorbidity. On the other hand, in co-morbid forms, the presence of SP does not appear to cause an amplification of the impairment. In fact, the level of the QoL in MDD, PD and GAD without SP is not worse than in the same three disorders in comorbidity with SP;
3. the data appear to produce some doubts about the hypothesis that SP may be an antecedent of more serious disorders, useful in identification in terms of prevention.

The study has undoubted limitations: firstly, the age of SP onset has not been studied and the different sub-types have not been identified. Furthermore, the transversal design makes it possible to generate hypotheses rather than to verify them. However, the same methodology by adopting the formula “Attributable Burden of Specific Disorder = SF-12 score in a matched sample of people without the disorder SF-12 score of people with diagnosis of this disorder” was proposed also in previous studies on the same database for measuring the attributable burden due to several disorders in worsening of QoL [27-33]. This method guarantees the homogeneity of the control groups without the specific disease analyzed, used as a comparison criterion to calculate the attributable burden due to a given disease or disorder, thus allowing a reliable comparison between the attributable burden of different diseases.

## CONCLUSION

An epidemiological study conducted by clinical interviewers through semi-structured interviews appears to re-dimension the impact of SP at least from the public health perspective. Future prospective studies will better clarify the role of SP in the context of anxiety disorders.

## Figures and Tables

**Table 1:** Lifetime Prevalence of Specific Phobia by sex and age.

-	**N (%)**	**χ^2^, 1df**	**p**	**OR**	**CI 95%**
<30 Males	1 (0.75)	---			
30-44	3 (0.79)	0.01°	0.999	0.95	0.04-10.34
45-64	6 (1.92)	0.24°	0.625	0.39	0.02-3.28
>64	2 (1.07)	0.01°	0.999	0.70	0.02-9.95
Total Males*	12 (1.18)	-	-	-	-
<30 Females	4 (2.90)	---	-	-	-
30-44	12 (2.67)	0.02	0.884	1.01	0.29-3.72
45-64	18 (3.73)	0.21	0.643	0.77	0.21-2.47
>64	8 (3.19)	0.02	0.875	0.91	0.22-3.34
Total Females*	42 (3.17)	10.10	<0.0001	2.74	1.38-5.52

*Females **vs.*.* Males °with Yates’s correction

**Table 2:** Comorbidity between Specific Phobia and Other Disorders.

-	**Comorb with** **Specific Phobia** **N (%)**	χ^2^*	**P**	**OR**	**CI 95%**
Major Depressive Disorder	9 (16.6)	20.72	<0.0001	4.82	2.12-10.62
Obsessive Compulsive Disorder	7 (7.4)	35.68	<0.0001	8.80	3.39-21.90
Panic Disorder	9 (16.6)	27.28	<0.0001	5.89	2.57-13.01
Social Phobia	2 (3.7)	11.34	<0.0001	17.53	2.29-105.0
Agoraphobia	5 (9.2)	23.79	<0.0001	10.03	3.19-29.38
General Anxiety Disorder	10 (18.5)	64.36	<0.0001	11.57	5.09-25.71
Post-Traumatic Stress Disorder	2 (3.7)	3.22	0.07	5.81	0.89-27.60
Eating Disorders	4 (7.4)	17.16	<0.0001	7.23	2.05-22.98
Anorexia	3 (5.5)	13.79	<0.0001	11.13	2.41-44.19
Bulimia	0 (0)	0.01	0.999	---	----
Binge Eating Disorder	1 (1.9)	0.024	0.621	4.29	0.20-33.44

*With Yates’ Correction

**Table 3:** Comorbidity between Specific Phobia and other disorders in young people.

**Disorders**	**Frequency** **<30 years** **N (%)**	**Frequency** **≥30 Years** **%**	χ^2^*	**P**	**OR**	** CI 95%**
At least one diagnosis (anxiety, mood or eating disorder) in comorbidity	5 (50)	47.7	0.001	0.999	1.1	0.2-5.2
Major Depressive Disorder	3 (30)	13.6	0.614	0.433	2.7	0.4-17.2
Panic Disorders	1 (10)	18.2	0.025	0.876	0.5	0.1-5.0
General Anxiety Disorder	2 (20)	18.2	0.001	0.999	1.1	0.1-7.7

*With Yates’ Correction

**Table 4:** Comparison on Quality of Life of people with Specific Phobia and people without Specific Phobia (matching controls ½ from the community sample drawn after standardization by gender and age).

**Diagnosis**	Specific Phobia*	Controls without diagnosis (½)*	**F** **(df)**	**p**
Specific Phobia without comorbidity (N=28)	38.3±5.2	38.7±5.4	0.059 (1,82,83)	0.809
Specific Phobia with comorbidity (N=26)	33.10±7.1	37.8±4.3	13.498 (1,78,79)	<0.0001
Specific Phobia (Total) (N=54)	35.8±6.1	38.3±4.9	7.927 (1,160,161)	0.005

**Table 5:** Attributable burden in worsening Quality of Life due to Specific Phobia and comparison with other disorders.

**Disorders**	**SF-12** **(mean±sd)**	**Attributable Burden due to Disorder**	**Comparison with Specific Phobia without comorbidity**	**Comparison with Specific Phobia with Comorbidity**
Celiac Disease	35.8±5.7	2.4±1.0 (N=60)	df 1,86,87 F= 81.208 p<0.0001	df 1,84,85 F=19.349 p<0.0001
Multiple Sclerosis	29.5± 7.3	7.0±3.5 (N=201)	df 1,227,228 F= 98.312 p<0.0001	df 1,234,235 F=10.355 p=0.001
Wilson’s Disease	33.8±9.0	4.4±1.7 (N=23)	df 1,49,50 F= 115.856 p<0.0001	df 1,49,50 F=0.123 p=0.728
Carotid Atheriosclerosis	30.6±8.1	6.2±5.0 (N=46)	df 1,72,73 F= 36.759 p<0.0001	df 1,70,71 F=1.861 p=0.177
Major Depressive Disorder	33.8±9.2	5.6±3.6 (N=37)	df 1,63,64 F= 102.370 p<0.0001	df 1,61,62; F=0.950 p=0.334
Eating Disorders	34. ±6.2	4.4±6.6 (N=60)	df 1,86,87 F= 10.135 p<0.0001	df 1,84,85 F=0.047 p=0.829
Panic Disorders	35.5±4.6	2.9±0.9 (N=123)	df 1,149,150 F= 175.987 p<0.0001	df 1,147,148 F=7.370 p=0.007
Obsessive Compulsive Disorder	35.4±6.9	2.9±6.0 (N=88)	df 1,114,115 F= 4.799 p<0.031	df 1,112,113 F=2.085 p=0.152
Post-Traumatic Stress Disorder	36.3±6.1	3.9±1.0 (N=26)	df 1,52,53 F= 183.224 p<0.031	df 1,50,51 F=1.078 p=0.304
Specific Phobia Without comorbidity	38.3±5.2	0.4±4.9 (N=28)		df 1,52,53 F= 33.855 p<0.031
Specific Phobia with comorbidity	33.10±7.1	4.7±3.8 (N=26)	df 1,52,53 F= 33.855 p<0.031	

**Table 6:** Worsening of Quality of Life in Specific Phobia in co-morbid disorders in comparison with disorders without Specific Phobia and Attributable Burden due to Specific Phobia.

-	**Major Depressive Disorder**	**Panic Disorders**	**Generalized Anxiety Disorder**
Age and Gender	+SP: 9 F 41.7±15.8 -SP: 18 F 41.9±14.9	+SP: 6 F + 3 M 50.3±19.9 -SP: 6 F + 3 M 50.8±19.3	+SP: 9 F + 1 M 48.8±17.4 -SP: 9 F +1 M 48.08±16.7
Co-morbid with SP	(N=9); SF-12 score: 33.0±9.1	(N=9); SF-12 score: 33.3±9.8	(N=10); SF-12 score: 30.9±7.4
Without SP (1/2)	(N=18); SF-12 score: 35.5±7.7	(N=18); SF-12 score: 34.55±6.2	(N=20); SF-12 score: 32.7±7.5
ANOVA Comparison	(df 1,25,26) F=0.633 p=0.434	(df 1,25,26) F=0.152 p=0.700	(df 1,28,29) F=0.349 p=0.560
Attributable Burden due to SP	2.5±5.7	1.2±3.6	1.8±4.9
Attributable Burden due to SP in people without comorbid conditions	(N=28); 0.4±4.9	(N=28); 0.4±4.9	(N=28); 0.4±4.9
ANOVA Comparison between SP burden in such disorder *vs.* people without comorbidity	(df 1,35,36) F=1.158 p=0.289	(df 1,35,36) F=0.203 p=0.655	(df 1,36,37) F=0.305 p=0.443

Legend SP: Specific Phobia F: female M: male
